# Renal Ciliopathies: Sorting Out Therapeutic Approaches for Nephronophthisis

**DOI:** 10.3389/fcell.2021.653138

**Published:** 2021-05-13

**Authors:** Marijn F. Stokman, Sophie Saunier, Alexandre Benmerah

**Affiliations:** ^1^Department of Genetics, University Medical Center Utrecht, Utrecht, Netherlands; ^2^Université de Paris, Imagine Institute, Laboratory of Inherited Kidney Diseases, INSERM UMR 1163, Paris, France

**Keywords:** hereditary kidney disease, ciliopathy, nephronophthisis, signaling, cell cycle, drug screen, therapy, gene therapy

## Abstract

Nephronophthisis (NPH) is an autosomal recessive ciliopathy and a major cause of end-stage renal disease in children. The main forms, juvenile and adult NPH, are characterized by tubulointerstitial fibrosis whereas the infantile form is more severe and characterized by cysts. NPH is caused by mutations in over 20 different genes, most of which encode components of the primary cilium, an organelle in which important cellular signaling pathways converge. Ciliary signal transduction plays a critical role in kidney development and tissue homeostasis, and disruption of ciliary signaling has been associated with cyst formation, epithelial cell dedifferentiation and kidney function decline. Drugs have been identified that target specific signaling pathways (for example cAMP/PKA, Hedgehog, and mTOR pathways) and rescue NPH phenotypes in *in vitro* and/or *in vivo* models. Despite identification of numerous candidate drugs in rodent models, there has been a lack of clinical trials and there is currently no therapy that halts disease progression in NPH patients. This review covers the most important findings of therapeutic approaches in NPH model systems to date, including hypothesis-driven therapies and untargeted drug screens, approached from the pathophysiology of NPH. Importantly, most animal models used in these studies represent the cystic infantile form of NPH, which is less prevalent than the juvenile form. It appears therefore important to develop new models relevant for juvenile/adult NPH. Alternative non-orthologous animal models and developments in patient-based *in vitro* model systems are discussed, as well as future directions in personalized therapy for NPH.

## Introduction

Nephronophthisis (NPH) is an autosomal recessive kidney disease that accounts for up to 15% of end-stage renal disease (ESRD) in children (Hamiwka et al., 2008; Salomon et al., 2009). In addition, homozygous deletions in the *NPHP1* gene, the most frequent cause of NPH (≈50% of genetically diagnosed cases), have recently been shown to be an important cause of renal disease in adults (Snoek et al., 2018).

Three clinical subtypes of NPH can be discerned. In juvenile NPH, the most prevalent form, ESRD develops at a median age of 13 years (Hildebrandt et al., 2009). Juvenile NPH is characterized by small to normal-sized kidneys that show marked tubulointerstitial fibrosis, thickened and disrupted tubular basement membranes and, in less than 50% of patients, corticomedullary cysts that arise from distal tubules in advanced stages of the disease (Hildebrandt et al., 2009). Patients present with a urine concentration defect leading to polyuria and polydipsia, anemia, proteinuria (in advanced stages) and progressive renal insufficiency (König et al., 2017; Stokman et al., 2018). The adult form of NPH is clinically and histologically similar to juvenile NPH and leads to adult-onset ESRD (>15 years old). This form is difficult to distinguish clinically from autosomal dominant tubulointerstitial kidney disease (ADTKD) which is also characterized by tubular damage, interstitial fibrosis and corticomedullary microcysts (Devuyst et al., 2019). In contrast, autosomal dominant polycystic kidney disease (ADPKD), which is more prevalent, is typically adult-onset with slow progression to ESRD [onset from 58 to 80 years (Chebib and Torres, 2016)] and characterized by enlarged kidneys with cysts distributed throughout the renal parenchyma (Harris and Torres, 2002).

In contrast to juvenile and adult NPH, infantile NPH leads to ESRD before the age of 5 years and typically presents with enlarged cystic kidneys, severe hypertension and a rapid disease course, making it difficult to distinguish from autosomal recessive polycystic kidney disease (ARPKD) based on renal symptoms only (Gagnadoux et al., 1989; Otto et al., 2003; Waters and Beales, 2011; Bergmann, 2018). Infantile NPH is characterized histologically by cortical cysts, absence of tubular basement membrane thickening and only moderate fibrosis (Gagnadoux et al., 1989; Salomon et al., 2009; Tory et al., 2009). This raises the question whether infantile NPH is really part of the NPH spectrum or rather represents a distinct subtype of cystic kidney disease.

Mutations in more than 20 different genes have been identified to cause NPH (Braun and Hildebrandt, 2017; Devlin and Sayer, 2019). Most of these genes encode proteins that localize to primary cilia and their disruption causes defects in cilia formation, length and/or composition. NPH therefore falls into the category of ciliopathies, a group of multisystemic disorders linked to primary cilia dysfunction. Primary (non-motile or sensory) cilia (PC) are microtubule-based antennae present on the surface of nearly all cell types in vertebrates during development and/or in adult tissues, from where they sense mechanical stress (shear stress), molecules (odor, light) and control key signaling pathways (Hedgehog, Wnt, TGFβ, etc.). PC dysfunction is therefore associated with a variety of phenotypes in different organs including kidney failure, retinal dystrophy, liver fibrosis, skeletal dysplasia, cerebellar vermis hypoplasia and obesity. In the kidney, PC are present on the apical surface of most tubular cells from where they integrate different stimuli during kidney homeostasis and function. The discovery of the ciliary localization and functions of polycystin-1 and polycystin-2, the products of the ADPKD genes *PKD1* and *PKD2*, led to the hypothesis that cilia control oriented cell division and proliferation of tubular cells, and that ciliary defects therefore result in cystic kidneys, which became a classic sign of ciliopathies.

In accordance with the role of PC in most organ systems, 10–20% of NPH patients have extrarenal manifestations defining syndromic forms, including Senior-Løken syndrome (NPH and retinal dystrophy), Bardet-Biedl syndrome (retinal dystrophy, obesity and polydactyly), Joubert syndrome (cerebellar vermis hypoplasia, hypotonia and developmental delay) and cranioectodermal dysplasia (narrow thorax and short stature) (Braun and Hildebrandt, 2017).

Current drug therapy for NPH is merely supportive, aimed at treatment of anemia, hypertension, growth retardation and other symptoms related to chronic kidney disease (CKD) (Braun and Hildebrandt, 2017). Although symptomatic treatment can prevent accelerated decline of renal function (for example due to hypertension) and improve quality of life for NPH patients, it does not target underlying disease mechanisms and ESRD cannot be prevented. The only curative treatment for NPH patients is a renal transplantation, as NPH does not recur in the transplanted kidney (Hamiwka et al., 2008; Tayfur et al., 2011).

Therapy for NPH-related ciliopathies has been an active field of research for more than 15 years. Next-generation sequencing techniques have accelerated gene discovery and uncovered numerous targetable genes and pathways. The most important drug targets for renal ciliopathies (i.e., NPH and PKD) have been reviewed by Molinari and Sayer (2017). Most of these drugs target mechanisms discovered in the context of ADPKD and were subsequently shown to also reduce cystogenesis in mouse or zebrafish models of (mostly infantile) NPH. Contrary to ADPKD, however, tubulointerstitial fibrosis is more important than cystogenesis in the pathophysiology of juvenile NPH. Consequently, aberrant DNA damage-response signaling and proinflammatory signaling, which have been proposed to contribute to fibrosis in juvenile NPH (Slaats et al., 2016), are interesting emerging targets and readouts. However, underlying disease mechanisms and potential targets for therapeutic interventions are not fully elucidated. Moreover, in contrast to ADPKD, thus far no clinical trial has been conducted in NPH patients.

If juvenile NPH is detected in an early disease stage, there is a therapeutic window of a few years in which treatment could delay or prevent renal replacement therapy. Previous reviews of therapies for renal ciliopathies focused on models of PKD and cystic (infantile) NPH or did not discern between renal and extrarenal manifestations. In this review we summarize the results from two main strategies for therapeutic intervention in NPH specifically that have been investigated in *in vitro* and *in vivo* model systems: pharmacotherapy (hypothesis-driven approaches including repurposed medication from PKD and untargeted drug screens) and gene-based therapy, in relation to the pathophysiology of different aspects of the NPH phenotype. Promising developments in personalized disease modeling and drug delivery are discussed, as well as challenges in clinical trials for NPH and orphan-drug development. Finally, we provide a rationale for future studies in this field.

## Pathophysiology of Nephronophthisis

### Genetic Basis

**FIGURE 1:**
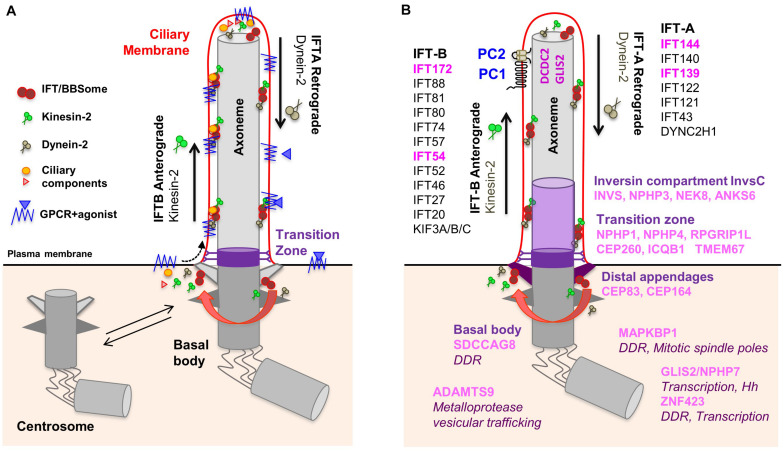
Composition of the primary cilium. **(A)** The mother centriole docks to the apical plasma membrane and forms the basal body which is connected to the plasma membrane by transition fibers. The basal body anchors the ciliary axoneme, consisting of 9+0 microtubule doublets. At the transition zone, Y-linkers connect the axoneme to the ciliary membrane. The transition zone functions as a gate through which membrane receptors and other proteins can enter the cilium. Intraflagellar transport (IFT) inside the cilium is mediated by kinesin-2 motors (anterograde transport) and dynein motors (retrograde transport). **(B)** NPH proteins localize to distinct functional compartments of the primary cilium. IFT proteins in pink are encoded by NPH-associated genes. No ciliary localization was demonstrated for ZNF423 and MAPKBP1.

Most ciliopathy-associated genes encode proteins that localize to PC which are assembled in quiescent cells (G_0_) from the centrosome, called ‘basal body’ when engaged in ciliogenesis (Figure 1A). During this process, the mother centriole docks onto cellular membranes through its distal appendages and then the distal ends of the nine centriolar microtubule doublets extend to form the ciliary axoneme which is surrounded by the ciliary membrane. The generation and maintenance of the unique molecular composition of the ciliary compartment (proteins and lipids) is based on the presence of the transition zone which acts as a gate at the base of the cilium and a highly conserved active transport machinery, the intraflagellar transport (IFT), which mediates in and out trafficking across this barrier through anterograde (IFT-B) and retrograde (IFT-A) transport along axonemal microtubules. The IFT and the BBSome, a protein complex encoded by genes mutated in Bardet-Biedl Syndrome, are also involved in the trafficking of signaling receptors enriched at the ciliary membrane such as G-protein coupled receptors (GPCRs) (Reiter et al., 2017).

Nephronophthisis is caused by mutations in 22 genes (full list in Table 1), which encode NPH proteins (NPHP). Of note, not all these genes have an official *NPHP* alias. Most NPHPs localize to and define three functionally important ciliary subdomains: the transition zone (NPHP1, NPHP4, IQCB1/NPHP5, CEP290/NPHP6 and RPGRIP1L/NPHP8), basal body/centrosome (SDCCAG8/NPHP10, CEP164/NPHP14, CEP83/NPHP18) and inversin compartment (Figure 1B) (Braun and Hildebrandt, 2017; Reiter et al., 2017; Devlin and Sayer, 2019). The inversin compartment (InvsC) comprises the products of four genes, *INVS/NPHP2, NPHP3, NEK8/NPHP9* and *ANKS6/NPHP16*, all of which have been implicated in infantile-onset NPH in addition to juvenile and/or adult NPH depending on the severity of the mutations (Gagnadoux et al., 1989; Olbrich et al., 2003; Otto et al., 2003, 2008; Bergmann et al., 2008; Tory et al., 2009; Hoff et al., 2013; Rajagopalan et al., 2015; Grampa et al., 2016). In addition to the above ciliary subdomains, NPH-causing mutations were also identified in genes encoding subunits of the IFT-A (*TTC21B/IFT139/NPHP12, WDR19/IFT144/NPHP13)* and IFT-B (*IFT172/NPHP17*, *TRAF3IP1/IFT54/SLS9*) sub-complexes (Bredrup et al., 2011; Davis et al., 2011; Halbritter et al., 2013a; Bizet et al., 2015). Importantly, NPH-related renal involvement was also reported in individuals harboring mutations in other IFT-A encoding genes (Perrault et al., 2012; Schmidts et al., 2013). This functional classification of NPH genes also corresponds to specific extrarenal manifestations. For example, genes encoding transition zone proteins are associated with retinal dystrophy, genes encoding InvsC proteins are associated with situs inversus and most genes encoding IFT subunits give rise to skeletal dysplasia.

**TABLE 1:** Localization of NPH proteins and associated phenotypes.

Ciliary localization Function	Gene	Locus	Extraciliary localization/function	NPH subtype(s)	Extrarenal features (Halbritter et al., 2013b; Braun and Hildebrandt, 2017; König et al., 2017; Luo and Tao, 2018)	References
Transition zone (TZ)	*NPHP1*	*NPHP1*	Cell junctions	Juvenile, adult	Retinitis pigmentosa, neurologic symptoms, liver disease	Hildebrandt et al. (1997); Saunier et al. (1997)
	*NPHP4*	*NPHP4*	Cell junctions Hippo signaling	Juvenile, adult	Retinitis pigmentosa, neurologic symptoms, congenital heart disease, liver disease (all infrequent)	Mollet et al. (2002); Otto et al. (2002)
	*IQCB1*	*NPHP5*	–	Juvenile, adult	Retinitis pigmentosa (in all patients), leber congenital amaurosis, neurologic symptoms	Otto et al. (2005); Estrada-Cuzcano et al. (2011)
	*CEP290*	*NPHP6*	DDR signaling	Infantile, juvenile, adult	Retinitis pigmentosa, leber congenital amaurosis, neurologic symptoms, liver disease	Sayer et al. (2006); Valente et al. (2006); Helou et al. (2007); Tory et al. (2007)
	*RPGRIP1L*	*NPHP8*	–	Infantile, juvenile, adult	Retinitis pigmentosa, neurologic symptoms, liver disease, polydactyly	Arts et al. (2007); Delous et al. (2007); Wolf et al. (2007)
	*TMEM67*	*NPHP11*	–	Infantile, juvenile, adult	Retinitis pigmentosa, neurologic symptoms, liver disease, polydactyly	Smith U. M. et al. (2006)
Intraflagellar transport complex IFT-B	*IFT172*	*NPHP17*	–	Infantile, juvenile, adult	Retinitis pigmentosa, neurologic symptoms, liver disease, skeletal anomalies, polydactyly	Halbritter et al. (2013a)
	*TRAF3IP1/IFT54*	*SLNS9*	Microtubule dynamics	Infantile, Juvenile	Retinitis pigmentosa, hepatic fibrosis, skeletal anomalies, obesity	Bizet et al. (2015)
Intraflagellar transport complex IFT-A	*TTC21B/IFT139*	*NPHP12*	Microtubule dynamics	Infantile, juvenile	Neurologic symptoms, situs inversus, liver disease, skeletal anomalies	Davis et al. (2011); Cong et al. (2014)
	*WDR19/IFT144*	*NPHP13*	–	Infantile, juvenile	Retinitis pigmentosa, liver disease (especially Caroli disease), pancreas anomalies, skeletal anomalies	Bredrup et al. (2011)
Inversin compartment	*INVS*	*NPHP2*	Cell junctions; mitotic spindle poles, midbody	Infantile, juvenile	Retinitis pigmentosa, neurologic symptoms, situs inversus, congenital heart disease, liver disease	Otto et al. (2003); O’Toole et al. (2006); Tory et al. (2009)
	*NPHP3*	*NPHP3*	–	Infantile, juvenile, adult	Retinitis pigmentosa, neurologic symptoms, situs inversus, congenital heart defect, liver disease	Omran et al. (2000); Olbrich et al. (2003); Bergmann et al. (2008); Tory et al. (2009)
	*NEK8*	*NPHP9*	DDR and Hippo signaling	Infantile, juvenile	Situs inversus, congenital heart disease, liver disease, pancreas anomalies	Otto et al. (2008); Grampa et al. (2016)
	*ANKS6*	*NPHP16*	–	Infantile, juvenile, adult	Neurologic symptoms, situs inversus, congenital heart disease, liver disease	Hoff et al. (2013); Taskiran et al. (2014)
Basal body	*SDCCAG8*	*NPHP10*	DDR signaling	Infantile, juvenile, adult	Retinitis pigmentosa, neurologic symptoms, obesity, hypogenitalism	Otto et al. (2010); Watanabe et al. (2019)
	*CEP164*	*NPHP15*	DDR signaling	Juvenile	Retinitis pigmentosa, Leber congenital amaurosis, neurologic symptoms, liver disease, polydactyly	Chaki et al. (2012)
	*CEP83*	*NPHP18*	–	Infantile, juvenile	Retinitis pigmentosa, neurologic symptoms, liver disease	Failler et al. (2014)
Near basal body	*ADAMTS9*	*NPHP21*	Metalloproteinase function, protein trafficking	Infantile, juvenile	Deafness, short stature, developmental delay	Choi et al. (2019); Nandadasa et al. (2019)
Ciliary axoneme	*GLIS2*	*NPHP7*	Nucleus, role in transcription	Juvenile	–	Attanasio et al. (2007)
	*DCDC2*	*NPHP19*	Microtubule dynamics, Wnt signaling	Juvenile	Liver disease, deafness	Schueler et al. (2015)
No ciliary localization	*ZNF423*	*NPHP14*	DDR signaling and transcription	Infantile	Neurologic symptoms, situs inversus	Chaki et al. (2012)
	*MAPKBP1*	*NPHP20*	DDR and JNK signaling; Mitotic spindle poles during mitosis	Juvenile, adult	Scoliosis, facial dysmorphisms^1^	Macia et al. (2017)

*Presence and severity of extrarenal features varies between patients. ^1^Tentative association in one and two families, respectively.*

Interestingly, several NPHPs have not been linked directly to ciliary functions including GLIS2 (*NPHP7*, see below), ZNF423 (*NPHP14*) and the recently discovered gene MAPKBP1 (*NPHP20*). However, ZNF423 has been shown to interact with CEP290 and its murine ortholog *Zfp423* was shown to be involved in the transcription of ciliary genes (Chaki et al., 2012; Hong and Hamilton, 2016). Mutations in *MAPKBP1* were identified in several individuals presenting with late onset juvenile or adult NPH (Macia et al., 2017; Schonauer et al., 2020; Shamseldin et al., 2020). *MAPKBP1* encodes a non-ciliary scaffold protein involved in JNK signaling that localizes to mitotic spindle poles (Macia et al., 2017) as well as to the centrosome (Schonauer et al., 2020). Loss of *MAPKBP1* causes increased DNA-damage response signaling as observed in other NPH subtypes [see section “DNA-Damage Response (DDR)”] but no ciliogenesis defects (Macia et al., 2017), indicating that this may represent a new cilia-independent subtype of isolated NPH.

Of note, NPH can be a feature of syndromic ciliopathies caused by mutations in genes currently not classified as *NPHP* genes (*BBS* genes for example), resulting in more than 90 genes associated with an ‘NPH-related ciliopathy’ (Braun and Hildebrandt, 2017). In addition, biallelic mutations in *XPNPEP3* and *SLC41A1* can cause an NPH-like phenotype (O’Toole et al., 2010; Hurd et al., 2013). These genes and their functions fall outside the scope of this review.

### Pathophysiology

#### Cilia-Associated Signaling Pathways

The combination of the transition zone at the base of the cilium which limits passive diffusion of molecules and the active transport of ciliary proteins mediated by the IFT/BBSome complexes generates a closed compartment that allows a concentration of signaling molecules and rapid signal transduction independent from rest of the cell body (Hansen et al., 2020). The primary cilium therefore acts as a cellular signaling hub for chemosensory (for example GPCR) and mechanosensory (for example polycystin-1/polycystin-2 receptor-ion channel complex) signaling (Spasic and Jacobs, 2017). PC have also been proposed as a source of a specific subtype of extracellular vesicles or ‘ectosomes’ which are likely acting as signaling messengers (Wood et al., 2013; Wang and Barr, 2016; Nager et al., 2017; Phua et al., 2017). Ciliary defects can disrupt these pathways, which in turn can impair kidney development and, more importantly in NPH, maintenance of kidney architecture and function. Cilia-dependent signal transduction pathways have been extensively reviewed elsewhere (Wheway et al., 2018; Anvarian et al., 2019). Specific cilia-associated signaling pathways are discussed in more detail in Section “Targeting Ciliary Signaling Pathways,” which summarizes pathways that have been targeted pharmacologically in the context of NPH and/or ADPKD including mTOR, Hedgehog (Hh) and Hippo pathways. Of note, there is mounting evidence for crosstalk between ciliary signaling pathways and DNA-damage response (DDR) signaling discussed below (Wang et al., 2020).

#### DNA-Damage Response (DDR)

Studies over the past 10 years pointed to an important functional relationship between several NPHP gene products and DDR signaling (Slaats and Giles, 2015; Zhang J. Q. J. et al., 2019). DDR signaling is involved in the detection of DNA damage and activation of appropriate cellular responses including cell cycle arrest and activation of DNA repair mechanisms. Chaki et al. (2012) proposed that disruption of DDR signaling and consequent impairment of cell-cycle checkpoint control early in development leads to depletion of progenitor cells and dysplastic phenotypes, while milder disruptions of DDR signaling associated with hypomorphic mutations lead to a slow accumulation of DNA damage over time resulting in degenerative phenotypes such as NPH. Since the discovery of the role of *ZNF423/NPHP14* and *CEP164/NPHP15* in DDR signaling, this functional link has been confirmed in NPH genes *CEP290/NPHP6*, *NEK8/NPHP9*, *SDCCAG8/NPHP10* and *MAPKBP1/NPHP20* (Otto et al., 2008, 2010; Chaki et al., 2012; Choi et al., 2013; Airik et al., 2014; Slaats et al., 2014, 2015; Slaats and Giles, 2015; Macia et al., 2017). Interestingly, analysis of primary kidney cells from *Cep290*-deficient mice showed increased double-strand breaks, replication fork asymmetry and reduced fork velocity suggestive of increased replication stress, and increased levels of cyclin-dependent kinases (CDKs). These effects could be rescued *in vitro* using CDK inhibitors (Slaats et al., 2015). *Cep164* knockdown in ciliated cells induced epithelial-to-mesenchymal transition (EMT) and upregulation of fibrosis-associated genes, further linking aberrant DDR signaling and S-phase arrest to tubulointerstitial fibrosis in NPH (Slaats et al., 2014). Correspondingly, *MAPKBP1*-deficient cells showed increased DDR, and kidney sections from *MAPKBP1*-related NPH patients revealed tubular atrophy and massive tubulointerstitial fibrosis (Macia et al., 2017).

#### Polarity

Next to nuclear functions, specific NPH-associated proteins have additional roles outside the cilium. For example, NPHP1, INVS and NPHP4 localize to and regulate cell-cell junctions (Donaldson et al., 2002; Nurnberger et al., 2002; Delous et al., 2009). NPHP1 and NPHP4 are required for timely tight junction formation and interact with the proteins PALS1 and PAR6 involved in establishment of epithelial cell polarity. Knockdown of *NPHP1* and *NPHP4* in MDCK cells resulted in disorganized, multi-lumen structures, and loss of cell polarity (Delous et al., 2009). Similar findings were obtained for other NPHPs in murine IMCD3 kidney cells suggesting a shared function in polarity and epithelialization (Sang et al., 2011; Ghosh et al., 2012). Interestingly, in the case of TRAF3IP1/IFT54, the observed polarity defect could be linked to an increased stability of cytoplasmic microtubules (see below) (Bizet et al., 2015). Finally, the loss of *Glis2/Nphp7* causes NPH in mice through EMT driven by the expression of EMT specific genes such as SNAIL (Attanasio et al., 2007; Kim et al., 2008; Li et al., 2011). Altogether, these data suggest that the crucial roles of NPHP proteins in polarity/epithelialization likely contribute to the tubular atrophy and loss of nephrons observed in juvenile NPH.

#### Inflammation and Senescence

Most recently, activation of inflammation was proposed to be involved in tubulointerstitial fibrosis in NPH. NPHP1 is part of a protein complex with the kinase LKB1 that regulates cilia-controlled secretion of the chemokine CCL2, and *Nphp1* silencing induced increased Ccl2 expression in kidney cells (Viau et al., 2018). Ccl2 has been associated with macrophage activation and disease progression in CKD (Cao et al., 2015). In addition, mononuclear infiltrates and inflammation have been described in animal models of NPH including *pcy*/*Nphp3* and *Glis2/Nphp7* mice (Omran et al., 2001; Kim et al., 2008). Interestingly, *Glis2/Nphp7* knockout mice were shown to have increased tubular cell senescence (Jin et al., 2020). Replicative senescence leads to secretion of pro-inflammatory cytokines and activation of the NF-kB pathway, eliciting an inflammatory response, increased senescence in neighboring cells and extensive kidney damage as demonstrated in a *Glis2* knockout mouse and in other mouse models of CKD (Acosta et al., 2013; Docherty et al., 2019; Jin et al., 2020). In addition, senescent tubular epithelial cells were demonstrated to be a source of pro-fibrotic Hh ligands (Jin et al., 2019). Similar processes could contribute to tubulointerstitial damage in other subtypes of juvenile NPH with known defects in DNA-damage response signaling and cell cycle progression, although it is unknown whether tubular cell senescence is a general feature of NPH.

#### Additional Mechanisms

Additional cilia-independent mechanisms that have been pharmacologically targeted in NPH, including glycosphingolipids and cholesterol, and microtubule dynamics, are discussed in Section “Pharmacotherapy.” Other factors were not directly targeted but have been proposed to play a role in the pathophysiology of NPH. For example, oxidative stress, the endoplasmic reticulum (ER) stress response and mitochondrial dynamics were shown to be dysregulated in the *jck* mouse model of NEK8/NPHP9 (Bracken et al., 2016). In addition, the NPH-associated metalloproteinase ADAMTS9 was involved in regulation of ER-Golgi vesicular transport (Yoshina et al., 2012). However, ADAMTS9 was also shown to localize in vesicular structures near the base of the cilium and to be required for ciliogenesis, a function which appears to be distinct from its role in ER-Golgi transport (Choi et al., 2019; Nandadasa et al., 2019). The relationship between the ubiquitin-proteasome system, autophagy and primary cilia has been recently reviewed (Boukhalfa et al., 2019; Wiegering et al., 2019). Finally, increased formation of ciliary extracellular vesicle has been observed in BBS mutant cells suggesting that dysregulation of this process might contribute to ciliopathies (Nager et al., 2017; Akella et al., 2020). However, for most proposed mechanisms it remains unclear how individual factors contribute to specific NPH phenotypes.

## Available Model Systems

### Animal Models

Although studies in animal models of NPH have provided above-mentioned insights into pathophysiology of NPH, most models do not recapitulate the juvenile NPH phenotypes.

Zebrafish larva (24–72 h post fertilization) is a widely used organism to study renal ciliopathies. This model is low cost, fast and efficient, allowing *in vivo* imaging, functional studies and large-scale screening of small molecules for therapeutic approaches. Most models have been based on morpholino-based knockdown of the respective *nphp* genes, resulting in loss of function like conditions, typically resulting in severe developmental defects including cysts in the proximal pronephros and/or defects in the distal part of the pronephros associated with ciliary defects (Slanchev et al., 2011; Song et al., 2016; Elmonem et al., 2018). It is unlikely that fibrosis could be monitored/observed in early developmental stages in the simple pronephros which is present in the embryo; this would likely require the development of adult fish models. Generation of mutant zebrafish lines to study more slowly progressive juvenile NPH is limited because most described mutants show either too severe phenotypes with early death at larval stages, or no phenotype in the adults (Song et al., 2016; Macia et al., 2017), likely due to compensation mechanisms (El-Brolosy and Stainier, 2017). Interestingly, a recent study described a hypomorphic mutation in *tmem67* (*NPHP11*), leading to ciliary defects and tubular cysts in adult zebrafish kidneys (Zhu et al., 2021), indicating that such models can be generated. Unfortunately, fibrosis was not monitored in this study.

Mouse models more accurately represent human kidney physiology and have been extensively used to characterize mechanisms underlying ciliopathies as well as CKD and associated fibrosis (Norris and Grimes, 2012; Ko and Park, 2013; Nogueira et al., 2017). However, while kidney fibrosis has been observed in mouse models of PKD and after toxic challenges, mice appear to be relatively resistant to NPH-related fibrosis. Indeed, published knockout mouse models corresponding to juvenile NPH including *Nphp1*, *Nphp4*, *Nphp5* and *Mapkbp1* do not show an obvious spontaneous renal phenotype (Jiang et al., 2008; Won et al., 2011; Ronquillo et al., 2016; Macia et al., 2017). Interestingly, only *Glis2/Nphp7*-knockout mice show a phenotype that fully represents juvenile NPH, comprising smaller kidneys with increased apoptosis, immune infiltration, fibrosis and tubular dilations on kidney sections, increased serum creatinine and polyuria (Kim et al., 2008).

Mouse models of InvsC defects (*Invs, pcy/Nphp3*, *jck/Nek8/Nphp9* and *Anks6/Nphp16*-mutated mice*)* show enlarged cystic kidneys characteristic of infantile NPH, although *pcy/Nphp3* and *Anks6/Nphp16* mice have slow disease progression and (late stage) tubulointerstitial fibrosis (Omran et al., 2001; Bakey et al., 2015). The *pcy/Nphp3* mouse model, which harbors a homozygous missense mutation in *Nphp3*, is characterized by cysts at the corticomedullary junction (later throughout the kidney), kidney enlargement and late-stage fibrosis (Takahashi et al., 1991; Omran et al., 2001; Olbrich et al., 2003). Consequently, *pcy/Nphp3* mice show more resemblance to ADPKD than to juvenile NPH. This is relevant because contrary to ADPKD, cyst formation in juvenile NPH likely occurs secondary to tissue degeneration and atrophy instead of being a driving mechanism, so targetable mechanisms in both conditions could diverge (Braun and Hildebrandt, 2017; Srivastava et al., 2018). Similarly, *jck/Nek8/Nphp9* mice, which show enlarged cystic kidneys and rapid disease progression, have been used as a model for (AR)PKD (Bukanov et al., 2006). These differences between human and animal phenotypes should be taken into account in pharmacological studies in NPH.

With the exception of juvenile NPHP genes mentioned above, genetic invalidation of most NPHP genes causes cystic kidney enlargement in mice (Table 2). These severe phenotypes could partly be explained by predominantly complete loss of function mutations in the mice models, which do not correspond to the (often hypomorphic) mutations in (at least) part of patients with juvenile NPH. The same limitations are found in the Lewis polycystic kidney (LPK), Wistar polycystic kidney (Wpk) and *Cy* rat models, with mutations in the orthologs of human *NEK8/NPHP9*, *TMEM67/NPHP11* and *ANKS6/NPHP16* genes, respectively. These all show rapidly progressive cystic kidney enlargement reminiscent of infantile NPH or ARPKD phenotypes (Cowley et al., 1993; Gattone et al., 2004; Brown et al., 2005; Smith U. M. et al., 2006; Phillips et al., 2007; McCooke et al., 2012; Taskiran et al., 2014).

**TABLE 2:** Rodent models of NPH discussed in this review.

Species	Name	Human ortholog	Type of mutation	Renal disease progression	Survival	Tubular phenotype	Interstitial phenotype	Urine concentration defect	Kidney size	Extrarenal phenotypes	References
Mouse	*Nphp1*	*NPHP1*	Out-of-frame deletion exon 20	NA	Normal	–	–	NA	Normal	Male infertility	Jiang et al. (2008)
	*Invs*	*INVS/NPHP2*	Out-of-frame deletion exon 3–11	Rapid (kidney failure around age 1 week)	Around 1 week	Absence of tubular atrophy and tubular basement membrane irregularities; corticomedullary cysts arising from proximal tubule and collecting duct;	–	NA	Enlarged	Situs inversus, biliary obstruction/atresia	Yokoyama et al. (1993); Lowe et al. (1996); Mochizuki et al. (1998); Morgan et al. (1998); Phillips et al. (2004)
	*Polycystic kidney disease (Pcy)*	*NPHP3*	Missense	Slow (kidney failure in adult mice)	NA	Tubular atrophy and tubular basement membrane thickening; corticomedullary cysts followed by entire kidney, tubular dilations arising predominantly from distal tubules and collecting duct	Tubulointerstitial fibrosis and inflammation (late stage)	NA	Enlarged	Cerebral vascular aneurysms	Takahashi et al. (1991); Omran et al. (2001); Olbrich et al. (2003)
	*Nphp4*	*NPHP4*	Missense	NA	Normal	–	–	NA	Normal	Retinal disease, male infertility	Won et al. (2011)
	*Iqcb1*	*IQCB1/NPHP5*	Gene trap leading to loss of function	NA	NA	–	–	NA	Normal	Retinal disease	Ronquillo et al. (2016)
	*Cep290*	*CEP290/NPHP6*	Gene trap inserted in intron 25 leading to premature stop codon	NA	129/Ola background: survival past age 1 year	Small cysts in cortex arising from collecting duct	–	Evidence of polyuria and polydipsia	NA	Retinal disease, cerebral abnormalities	Hynes et al. (2014)
			Rd16: deletion of exons 35 to 39	NA	NA	–	–	NA	Normal	Retinal disease	Chang et al. (2006)
			Knockout	Slow	Mixed C57BL/6 and 129/SvJ background: 80% lethality within first weeks due to hydrocephalus; surviving mice lived until age 2 years	Corticomedullary cysts develop after 12 months	–	NA	Enlarged	Retinal disease, cerebral and cerebellar abnormalities	Rachel et al. (2015)
			Gene trap inserted in intron 25 leading to premature stop codon	Rapid	C57BL/6 and 129/SvJ backgrounds: majority dies prenatally; mice that survive to age 2–3 weeks show severe cystic kidney disease	Loss of tubules (not further specified); corticomedullary cysts	Cellular infiltrate	NA	Enlarged	Retinal disease, hepatic pallor	
	*Glis2*	*GLIS2/NPHP7*	Knockout	NA	>40% lethality by age 10 months	Atrophy of proximal tubules, minimal tubular basement membrane thickening; corticomedullary and glomerular cysts	Tubulointerstitial inflammation and fibrosis starting at age 8 weeks	NA	Reduced	–	Attanasio et al. (2007); Kim et al. (2008)
	*Rpgrip1l (Ftm)*	*RPGRIP1L/NPHP8*	Knockout	NA	Embryonically lethal	Cortical microcysts arising from proximal tubule at 18.5 days post conception	–	NA	NA	Exencephaly, microphthalmia, situs inversus, liver abnormalities, polydactyly	Delous et al. (2007)
	*Juvenile cystic kidneys (Jck)*	*NEK8/NPHP9*	Missense	NA	100% lethality by age 25 weeks in males and age 80 weeks in females	Basement membrane disruptions; corticomedullary cysts followed by entire kidney, arising from collecting duct, in later stage also from distal tubule and loop of Henle	–	NA	Enlarged	–	Atala et al. (1993); Liu et al. (2002); Smith L. A. et al. (2006); Otto et al. (2008)
			Knockout	NA	Die within hours after birth due to congenital heart defect	Proximal tubule dilation; glomerular cysts and (few cysts develop in kidney explant culture)	–	NA	Normal	Situs inversus, cardiac anomalies	Manning et al. (2013)
	*Sdccag8*	*SDCCAG8/NPHP10*	Gene trap leading to loss of function	Slow	Survival > 250 days	Initial cortical cysts followed by corticomedullary cysts, arising from distal convoluted tubule and collecting duct; glomerular cysts	Tubulointerstitial fibrosis (late stage)	NA	Enlarged (late stage)	Retinal disease	Airik et al. (2014)
	*Bilateral polycystic kidneys (Bpck)*	*TMEM67/NPHP11*	Deletion	Rapid (kidney failure around age 3 weeks)	Survive to age 3 weeks	Corticomedullary cysts arising from distal tubule and collecting duct, later mild dilation of some proximal tubules	–	NA	Enlarged	Hydrocephalus, spermatogenesis defects	Cook et al. (2009)
	*Ttc21b (Thm1)*	*TTC21B/NPHP12*	Loss of function mutation	NA	Embryonically lethal	Cystic dilations of glomeruli, proximal tubules and ascending loops of Henle	–	NA	NA	–	Tran et al. (2008, 2014)
			Conditional *Ttc21b* knockout using ROSA26Cre ERT+	Rapid (cystic kidney disease and elevated BUN by age 6 weeks when *Ttc21b* is inactivated before P12-14)	NA	Cortical cysts arising from proximal tubule, loop of Henle and collecting duct	–	NA	Enlarged	–	Tran et al. (2014)
	*Cep164*	*CEP164/NPHP15*	Kidney-specific *Cep164* inactivation using Hoxb7-Cre (collecting duct)	Rapid (kidney failure around 3 weeks)	Median survival 25 days	Cysts in medulla followed by cortex and entire kidney, arising from collecting duct	–	NA	Enlarged	–	Airik et al. (2019)
	*Anks6*	*ANKS6/NPHP16*	Missense mutation	Slow	Survival to age 18 months	Cysts arising from collecting duct, thick ascending limb of loop of Henle, to a lesser extent from distal tubule; glomerular cysts	Interstitial fibrosis	NA	Enlarged	–	Bakey et al. (2015)
	*Mapkbp1*	*MAPKBP1/NPHP20*	Knockout	NA	Normal	–	–	NA	Normal	–	Macia et al. (2017)
	*Ofd1*	*OFD1*	Kidney-specific *Ofd1* inactivation using Ksp-Cre (distal tubule and collecting duct)	Severely impaired kidney function at age 1–3 months	Animals sacrificed at age 3 months	Cysts in medulla followed by cortex, arising from distal tubules; later glomerular cysts and proximal tubular cysts	–	NA	Enlarged	–	Zullo et al. (2010)
	*Ahi1*	*AHI1*	Knockout	Slow (kidney function impairment at age 1 year or older)	80% did not survive into adulthood	Tubular basement membrane disruption and thickening; corticomedullary microcysts and tubular dilations, mainly arising from proximal tubule	Interstitial cell infiltrate and fibrosis	Urinary concentration defect	Reduced by age 5 months	–	Lancaster et al. (2009)
Non-orthologous mouse models	*Fan1*	*FAN1*	Knockout mice treated with 2 mg/kg cisplatin	Rapid (kidney failure within 5 weeks after start of treatment)	NA	Tubular basement membrane thickening; tubular dilation; karyomegalic nuclei in proximal tubule	Tubulointerstitial inflammation and fibrosis	NA	NA	Bone marrow failure	Airik et al. (2016)
	*Prkar1a*	*PRKAR1A*	Kidney-specific *Prka1a* inactivation using Pkhd1-Cre (collecting duct)	NA	Animals sacrificed at age 3 months	Small cysts primarily arising from distal tubule and collecting duct	Interstitial fibrosis	NA	Normal (enlarged in 2/16 mice)	–	Ye et al. (2017)
	*Lkb1*	*STK11/LKB1*	Kidney-specific Lkb1 inactivation using Ksp-Cre (distal tubule and collecting duct)	Rapid (kidney failure around age 5 weeks)	50% survival around age 11 months	Tubular basement membrane thickening, tubular dilation, corticomedullary cysts at late stage	Tubulointerstitial inflammation and fibrosis	Impaired urine concentration at age 5 weeks	Reduced size at age 5 weeks	–	Viau et al. (2018)
	*Aatf*	*AATF*	Kidney-specific *Aatf* inactivation using Ksp-Cre (distal tubule and collecting duct)	Rapid (kidney failure around age 10 weeks)	Survival 10–15 weeks	Tubular basement membrane disruption and thickening; corticomedullary cysts arising from distal tubule, later glomerular cysts	Interstitial fibrosis	Urinary concentration defect	Reduced size at age 10 weeks	–	Jain et al. (2019)
Rat	Lewis polycystic kidney (LPK)	*NEK8/NPHP9*	Missense	Slow (kidney failure around 12–24 weeks)	No survival beyond age 26 weeks	Corticomedullary cysts, predominantly arising from collecting duct	Tubulointerstitial inflammation and fibrosis	NA	Enlarged	–	Phillips et al. (2007); McCooke et al. (2012)
	Wistar polycystic kidney (Wpk)	*TMEM67/NPHP11*	Missense	Rapid (kidney failure around age 3 weeks)	NA	Cysts in proximal tubule and collecting duct	–	NA	Enlarged	Cerebral abnormalities, hypoplastic spleen	Gattone et al. (2004); Smith U. M. et al. (2006)
	*Cy*	*ANKS6/NPHP16*	Missense	Homozygous: rapid (kidney failure around age 3 weeks)	Survive to age 3 weeks	Cysts in cortex and outer medulla	–	NA	Enlarged	–	Cowley et al. (1993); Brown et al. (2005)
			Missense	Heterozygous: slow	Males die of kidney failure within 1 year, females survive past 1 year	Thickened tubular basement membranes; dilatations of proximal and distal tubule and collecting duct	Tubulointerstitial fibrosis and inflammation	NA	Moderately enlarged	–	Cowley et al. (1993); Brown et al. (2005)

*Severity of the phenotype can depend on background strain. Presence of a renal phenotype was not reported for mouse models of *WDR19/NPHP13, ZNF423/NPHP14, IFT172/NPHP17, DCDC2/NPHP19* and *ADAMTS9/NPHP21* (Alcaraz et al., 2006; Cheng et al., 2007; Gorivodsky et al., 2009; Kern et al., 2010; Wang et al., 2011; Ashe et al., 2012; Dubail et al., 2014; Nandadasa et al., 2015; Schueler et al., 2015; Casoni et al., 2017). For *CEP83/NPHP18*, a cortical radial glial progenitor cell-specific conditional knockout was created that did not permit study of renal phenotypes (Shao et al., 2020). NPH can be a feature of other syndromic ciliopathies. Corresponding mouse models were not included in this table unless they were discussed in the main text. NA, not available/not reported.*

It is important to note that the type of genetic defect in animal models (knockout/loss of function or missense mutations) and the genetic background strain with associated genetic modifiers impact the severity of the phenotype and the utility of the model for human disease, as was demonstrated in mouse models of *Cep290*-associated ciliopathies (Ramsbottom et al., 2015, 2020). Patient-derived *in vitro* systems, for example urinary renal epithelial cells (URECs), kidney tubuloids and organoids, could partly solve these issues (Schutgens et al., 2019; Steichen et al., 2020).

### Kidney Organoids and Tubuloids

Primary kidney tubular epithelial organoids, or ‘tubuloids,’ can be efficiently established from adult stem cells derived from kidney biopsy material and from URECs. Tubuloids were employed to study infectious kidney disease in a personalized fashion and test the efficacy of cystic fibrosis treatment using a forskolin (cAMP) swelling assay, illustrating its utility in (personalized) drug screening (Schutgens et al., 2019). In contrast to tubuloids, which are restricted to epithelial tubular cells, kidney organoids were generated from human induced pluripotent stem cells (iPSC) that contained different parts of the nephron (glomerulus, proximal and distal tubule) and interstitial cells (Yousef Yengej et al., 2020). While these organoids lack collecting ducts, other protocols were recently setup to generate ureteric bud-derived collecting ducts enriched organoids (Kuraoka et al., 2020; Uchimura et al., 2020). Kidney organoids were also generated from adult differentiated kidney cells which incorporated extracellular matrix (ECM) cells, making them suitable to study interstitial phenotypes (Takasato et al., 2015; Ding et al., 2020). Although kidney organoids have mainly been studied as models of cyst development (ADPKD and ARPKD), with forskolin treatment inducing cystogenesis (Cruz et al., 2017; Low et al., 2019; Kuraoka et al., 2020), they have also been used to monitor fibrosis and myofibroblasts expansion upon IL-1β treatment (Lemos et al., 2018).

Limitations are that kidney organoids do not recapitulate the overall kidney morphology and do not take into account interactions between organs including vascularization of the glomeruli (unless they are transplanted into a host organism), an essential step required for generation of intratubular flow. In addition, only one study reported the generation of iPSCs-derived kidney organoid in the context of an NPH-related condition. The generated organoids were used as a source of tubular epithelial cells in which transciptomic and functional analyses confirmed major defects in polarity (Forbes et al., 2018), as previously reported for other NPHP genes in kidney epithelial cell lines (see section “Polarity”). However, these organoids did not show a cystic phenotype (Forbes et al., 2018). Further improvement of NPH patient-derived kidney organoids is required before these can be used in drug tests.

## Pharmacotherapy

### Hypothesis-Driven Therapies

In NPH, pharmacological targeting of the defective gene product is challenging because NPH-associated mutations typically lower expression of the protein and/or result in expression of a truncated protein. This is especially the case for homozygous deletion of *NPHP1*, the most frequent genetic event in NPH. Possibilities for targeted therapies therefore include enhancing stability/function of remaining mutated NPHP protein and/or of the complex in which it functions, or targeting the signaling pathway in which the protein plays a role, downstream pathophysiological mechanisms or modifier gene products (Kim and Kim, 2019).

Despite efforts to uncover specific approaches, many drugs investigated for the treatment of NPH were discovered in animal models of other renal ciliopathies, mainly ADPKD. ADPKD is the most prevalent inherited kidney disease and has a partly shared pathophysiology with NPH (cysts and fibrosis). As a result, important findings in the ADPKD field may be relevant for NPH.

Of note, some therapies listed below are aimed at prevention of NPH manifestations. While this approach in animal models could provide important insights, in practice most patients are symptomatic upon diagnosis therefore precluding any preventive treatment until the identification of relevant biomarkers in asymptomatic individuals.

#### Targeting Ciliary Signaling Pathways

##### Targeting GPCR/cAMP signaling

G-protein coupled receptors are a large family of signaling receptors, many of which localize to the ciliary membrane (Mykytyn and Askwith, 2017). Examples include odor receptors in olfactory neurons, rhodopsin in photoreceptors and smoothened (SMO) in various tissues. Key ciliary GPCRs are positively coupled to adenylate cyclases (AC) through GαS and their activation leads to production of cyclic AMP (cAMP), a key signaling intermediate which was shown to drive renal cyst formation in ADPKD and other renal ciliopathies (Mangoo-Karim et al., 1989; Ye and Grantham, 1993; Ghosh et al., 2012). Indeed, polycystin-2 was shown to interact with and inhibit AC while activating phosphodiesterase activity both resulting in a decreased amount of cAMP. In addition to the loss of this negative regulation in PKD conditions, dysregulation of calcium signaling may also contribute to elevated levels of cAMP through overactivation of ciliary AC and cAMP production leading to increased downstream signaling and cell proliferation (Sussman et al., 2020). Interestingly, NEK8/NPHP9 was shown to positively regulate polycystin-2 expression and ciliary localization (Smith L. A. et al., 2006; Sohara et al., 2008; Manning et al., 2013), in agreement with the cystic kidneys observed in infantile NPH linked to mutations in InvsC genes.

**FIGURE 2:**
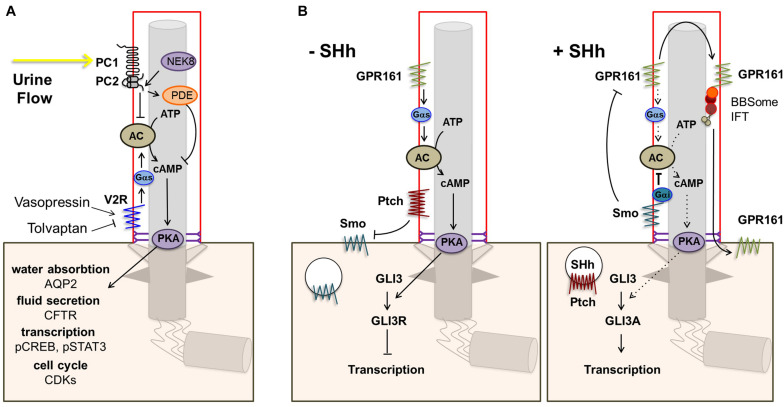
Ciliary GPCR signaling. **(A)** The vasopressin receptor type 2 (V2R) is located in the ciliary membrane. When activated, signal transduction via Gs alpha subunit (Gαs) leads to increased production of cAMP by adenylate cyclase (AC) and subsequent activation of protein kinase A (PKA). PKA phosphorylates aquaporin 2 (AQP2), which translocates to the apical cell membrane leading to increased water reabsorption. In addition, PKA phosphorylates the cystic fibrosis transmembrane conductance regulator (CFTR), leading to transport of Cl^–^ ions and fluid secretion into the lumen, and CREB and STAT3, enhancing transcription. Other downstream effects include increased CDK signaling. Opening of the polycystin-1/polycystin-2 (PC1/PC2) complex in response to urine flow inhibits the cAMP/PKA pathway via inhibition of AC5/6 and through PDE4C. **(B)** Other main ciliary GPCRs are Smo and the constitutively active Shh receptor GPR161. In the absence of Shh, Ptch1 excludes and inhibits Smo, leading to repression of Gli transcription factors by SuFu, and GPR161 signaling leads to increased cAMP-dependent activation of PKA. Elevated PKA further represses Gli transcription factors. Binding of a Hh ligand leads to entry of Smo and consequent removal of GPR161 from the ciliary membrane, resulting in decreased cAMP/PKA activity and activation of Gli transcription factors.

One of the main targetable sources of cAMP is the vasopressin receptor type 2 (V2R) encoded by *AVPR2*. V2R is an important GPCR in renal physiology that localizes to the basolateral membrane and to cilia of renal tubular epithelial cells (Sherpa et al., 2019). Activation of V2R by vasopressin leads to cAMP-dependent activation of PKA which in turn phosphorylates aquaporin 2 (AQP2), causing its translocation to the apical membrane where it controls water reabsorption, and upregulates AQP2 transcription (Figure 2A) (Nielsen et al., 2002). Disruption of this pathway could explain the urine concentration defect observed in NPH which partially mimics nephrogenic diabetes insipidus caused by mutations in *AVPR2* (Rosenthal et al., 1992; Van den Ouweland et al., 1992). Indeed, AQP2 targeting to the apical membrane in response to vasopressin was shown to be defective in human collecting duct cells in the context of Bardet-Biedl syndrome (Marion et al., 2011). Alternatively, decreased water reabsorption could result from disruption of tubular architecture and medullary osmotic gradient through defects in cell junctions and fibrosis as in other CKD (Krishnan et al., 2008; Bockenhauer et al., 2010).

In addition to its role in water reabsorption, V2R was involved in cystic kidney diseases (PKD). Its activation was shown to stimulate cyst growth through several cAMP-mediated mechanisms including activation of PKA leading to Cl^–^-dependent fluid secretion by cystic cells, and extracellular signal-regulated protein kinase (ERK) pathway-induced epithelial cell proliferation (Yamaguchi et al., 2000; Reif et al., 2011; Sherpa et al., 2019). The use of V2R antagonists such as tolvaptan, which inhibits V2R signaling reducing cAMP levels in renal collecting duct cells (Figure 2A), has proven to be a successful strategy in ADPKD. This approach was first shown to markedly reduce renal cyst formation and fibrosis and limit disease progression in a polycystic kidney rat model of ARPKD (PCK) and the *pcy/Nphp3* mouse model (Gattone et al., 2003). The V2R antagonist OPC31260 was subsequently shown to mitigate cystogenesis and renal function decline in a mouse model of ADPKD (Torres et al., 2004). Tolvaptan was shown to be effective in clinical trials (Torres et al., 2012, 2017, 2018) and is the first drug registered for treatment of adult ADPKD patients below 55 years of age and at risk of rapidly progressive disease as defined by clinical and/or genetic criteria (Chebib et al., 2018). There are no clinical trials registered for use of Tolvaptan in patients with NPH and there is only one case report in which Tolvaptan is described for the treatment of *NPHP3*-related infantile NPH without an effect on the very rapid renal function decline in this specific severe case (Strong et al., 2018). Such an approach could theoretically be used in the case of cystic infantile NPH, although the therapeutic window is small (mean age under 5), while its relevance in juvenile NPH is less apparent. Indeed, the presence of cysts is a late event in the juvenile/adult form and the potential role of cAMP remains to be investigated.

Another class of drugs that modulate renal cAMP levels by binding to GPCRs are somatostatin analogs, which activate somatostatin receptors and inhibit AC through the inhibitory G protein Gαi (Günther et al., 2018). Somatostatin analogs such as octreotide are used in the treatment of neuroendocrine tumors as inhibitors of growth hormone and several pancreatic and gastrointestinal hormones. In the context of kidney disease, octreotide was first shown to be well-tolerated and slow renal volume expansion in a 6-month trial with 14 adult patients with ADPKD (Ruggenenti et al., 2005). Octreotide was later shown to inhibit hepatic cyst development in the PCK rat model and reduce liver volume in *post hoc* analysis of the above-mentioned study in ADPKD patients (Masyuk et al., 2007; Caroli et al., 2010). It also reduced kidney volume in patients with ADPKD compared to placebo-treated patients (Hogan et al., 2010). In the context of NPH, octreotide was shown to significantly decrease cAMP levels and to rescue cilia defects and abnormal 3D spheroid phenotypes in *Nphp3*, *Cep290/Nphp6* and *Rpgrip1l/Nphp8* knockdown IMCD3 cells (Ghosh et al., 2012). These results suggest a positive effect of treatment with somatostatin analogs in NPH, although results have not been replicated in an animal model. Similarly, targeting the calcium-sensing receptor (CasR), a GPCR expressed in renal tubular cells, also leads to decreased production of cAMP through inhibition of AC (Riccardi and Valenti, 2016). Administration of the calcimimetic R-568 CasR agonist was shown to decrease cystogenesis and renal fibrosis in *pcy/Nphp3* mice (Chen et al., 2011). The above examples clearly show that decreasing cAMP levels is an effective therapeutic option in models of cystic kidney disease including ADPKD and (mostly infantile) NPH. The relevance of targeting cAMP in the context of juvenile NPH remains an open question as cystogenesis through proliferation and fluid secretion could be a less critical mechanism in this form of NPH (Srivastava et al., 2018).

A class of bioactive lipid metabolites called cyclooxygenase (COX) oxylipins (e.g., prostaglandins, leukotrienes) has been shown to be elevated in diseased kidneys of rodent models of NPH (*pcy*/*Nphp3* and *jck/Nek8/Nphp9* mice and Han:SPRD-*Cy/Anks6/Nphp16* rats) and other cystic kidney diseases (Ibrahim et al., 2014; Yamaguchi et al., 2014; Monirujjaman et al., 2017). A flax oil-enriched diet effectively reduced levels of COX-derived oxylipins compared to a control diet and mitigated interstitial fibrosis and cyst growth in *pcy/Nphp3* mice (Sankaran et al., 2004; Yamaguchi et al., 2015). In addition, treatment with COX inhibitors or dietary soy protein slowed disease progression in a heterozygous Han:SPRD-*Cy/Anks6/Nphp16* rat model (Sankaran et al., 2007; Ibrahim et al., 2014, 2015). Interestingly, the COX inhibitor Ibuprofen is used to treat patients presenting with Bartter Syndrome which is characterized by water and salt loss associated with elevated urinary prostaglandin E2 (PGE2). However, treatment of PCK rats with a COX inhibitor did not show any improvement of the cystic index or of kidney function (Sussman et al., 2020).

Although the exact role of COX-derived oxylipins in CKD remains unclear, PGE2 is known to exert its effect partly through increasing levels of cAMP through activation of tubular EP2/4 receptors (Elberg et al., 2007; Monirujjaman et al., 2017). The GPCR EP4 is one of the PGE2 receptors which control water and salt reabsorption (Li et al., 2018). Interestingly, EP4, which is also involved in AQP2 trafficking and could functionally compensate for the loss of V2R (Li et al., 2009), is present at cilia where it is involved in cAMP production and positive regulation of ciliogenesis through positive regulation of IFT (Jin et al., 2014; Jiang et al., 2019). It has however to be noticed that PGE2 acts through activation of four distinct receptors (EP1-4, see above) which control opposite downstream signaling pathways, complicating the understanding of the role of PGE2 in renal ciliopathies. Indeed, a recent study indicate that while PGE2 is pro-cystogenic *in vitro*, inhibition of EP2 and EP4 with antagonists resulted in a more severe disease in a *Pkd1* model due to increased inflammation (Lannoy et al., 2020). Even if among oxylipins PGE2 is an interesting candidate in the context of NPH (see above), evidence for increased or decreased production of PGE2, or of any other COX-derived oxylipins, in NPH patients is lacking so far.

##### mTOR inhibition

Next to cAMP signaling, an important signaling pathway that is inappropriately activated in ciliopathies is the mammalian target of rapamycin (mTOR) which functions as a central regulator in cellular metabolism, growth, proliferation, cell cycle and survival. It has been shown that cilia-dependent flow sensing negatively regulates mTORC1 through the kinase LKB1 (Boehlke et al., 2010). In ciliopathies like PKD, flow sensing is altered leading to mTOR pathway activation and uncontrolled cell growth and proliferation which induces cystogenesis (Lai and Jiang, 2020).

Administration of the mTOR pathway inhibitor rapamycin was shown to decrease proliferation, cystogenesis, kidney enlargement and renal function decline in heterozygous Han:SPRD-*Cy/Anks6/Nphp16* rats (Tao et al., 2005). In addition, ADPKD patients who had received kidney transplants were observed to have reduced volumes of affected kidney and liver if an mTOR inhibitor was included in the immunosuppressive regimen (Shillingford et al., 2006; Qian et al., 2008). However, 18-month rapamycin treatment of adults with early stage ADPKD did not halt disease progression and a clinically effective dose could not be achieved without systemic adverse effects (Serra et al., 2010; Shillingford et al., 2012). A study in zebrafish with morpholino-based knockdown of ciliopathy-associated genes including *nphp2, nphp5, nphp6* and *nphp11* provided additional evidence for the potential efficacy of mTOR inhibition in NPH-related ciliopathies. Treatment with rapamycin restored renal size and morphology in all morphants and partially rescued renal filtration decrease (Tobin and Beales, 2008). Treatment of *pcy/Nphp3* mice with rapamycin did not prevent initial cyst development but significantly reduced cyst enlargement, fibrosis and renal insufficiency (Gattone et al., 2009). This suggests an application in late-stage progression of NPH, the stage in which NPH is typically detected, if future targeted delivery can prevent systemic side effects of rapamycin.

##### Modulators of Hedgehog signaling

The ciliary GPCRs SMO and GPR161 are key players in the Hh signaling pathway, which is essential in embryonic development and adult tissue homeostasis and regeneration (Kopinke et al., 2020). In the absence of Hh, patched (PTCH1) localizes to the ciliary membrane and excludes and inhibits SMO. In addition, constitutive GPR161 activity in the cilium stimulates AC leading to increased cAMP-dependent activation of PKA which further leads to repressor forms of the GLI3 transcription factor (GLI3R). Upon binding of Hh to PTCH1, SMO enters the cilium whereas GPR161 is removed from the ciliary membrane, leading to decreased cAMP/PKA activity and activation of GLI3 (GLI3A; Figure 2B).

Hh signaling is involved in kidney development and preservation of the differentiated state of renal tubular epithelial cells (Li et al., 2011). Interestingly, Hh signaling has been implicated in fibrosis in different tissues through involvement in proliferation of myofibroblasts which expand in fibrotic tissues and secrete ECM components (Distler et al., 2019). In the kidney, Hh was shown to be produced in high amount by tubular cells in different models of kidney injury, stimulating interstitial mesenchymal cells which leads to fibrosis (Tan et al., 2016). In contrast, activation or downregulation of Hh signaling did not affect cyst formation in a *Pkd1* mouse model (Ma et al., 2019). NPHP proteins were shown to play important roles in Hh signaling including RPGRIP1L and ZNF423 as well as IFT involved in the transport of GPR161 and GLI transcription factors in and out of cilia (Vierkotten et al., 2007; Qin et al., 2011; Hong and Hamilton, 2016). Indeed, the Hh pathway has been shown to be dysregulated in numerous ciliopathies (Ruiz-Perez et al., 2007; Aguilar et al., 2012). Of note, a CRISPR-based screen to identify regulators of Hh signaling detected among many ciliopathy genes few genes mutated exclusively in NPH, not including *NPHP1* and *NPHP4* or the genes encoding InvsC proteins, suggesting that disrupted Hh signaling plays a less prominent role in isolated NPH than in other ciliopathies (Breslow et al., 2018).

A *Cep290*-gene trap mouse model of Joubert syndrome revealed increased Gli3 repressor isoform in the kidney, in agreement with decreased Hh signaling. Modulation of this pathway with the Hh agonist purmorphamine reconstituted impaired spheroid formation in collecting duct cells from *Cep290*-mutant mice and in URECs derived from a patient with Joubert syndrome (Hynes et al., 2014) as well the elongated cilia phenotype (Srivastava et al., 2017). In contrast, inhibition of Hh signaling using GLI and SMO small molecule antagonists Gant61 and Sant2 respectively prevented cyst formation in kidney explants from *Ttc21b/Ift139*-conditional knockout, *jck/Nek8/Nphp9* and *Pkd1* mutant mice (Tran et al., 2014). In contrast to CEP290, TTC21B/IFT139 is a negative regulator of Hh signaling (Tran et al., 2008), similar to other IFT-A subunits which control ciliary transport of GPR161 (Kopinke et al., 2020). The impact of ciliopathies on Hh signaling is therefore complex and appears to be gene and tissue dependent and may rely on its impact on the level of ciliary cAMP production. Although *in vitro* modulation of Hh signaling yielded promising results, pharmacological studies in patients are unlikely to be executed because of the risk of serious side effects including medulloblastoma associated with Hh stimulation (Molinari and Sayer, 2017).

##### YAP inhibition

The Hippo pathway controls numerous biological processes and is an important modulator of cell proliferation and organ size (Müller and Schermer, 2020). NPHP4 represses the Hippo signaling pathways through inhibition of LATS1/2-mediated phosphorylation of YAP and TAZ transcription factors, thereby stimulating transcription of target genes and cell proliferation (Habbig et al., 2011). In addition, InvsC proteins including NEK8/NPHP9 and NPHP3 form a complex that controls nuclear translocation of YAP/TAZ and transcription of target genes (Habbig et al., 2012; Frank et al., 2013). Dysregulation of the Hippo pathway can cause antiproliferative signaling resulting in hypodysplastic phenotypes and loss of differentiation, or increased proliferation leading to cystic phenotypes depending on the type of mutation (Habbig et al., 2011; Grampa et al., 2016; Müller and Schermer, 2019; Xu et al., 2020).

Transient *Nek8* knockdown in mIMCD3 cells resulted in increased YAP activation and enlarged spheroids, which could be rescued by administration of Verteporfin, an inhibitor of YAP transcriptional activity. Verteporfin additionally rescued pronephric cysts in zebrafish embryos overexpressing human NEK8 (Grampa et al., 2016). Verteporfin also reduced fibrosis in the unilateral ureteral obstruction mouse model (Szeto et al., 2016). Interestingly, Hippo components *Mst1/Mst2* double knockout mice presented with an NPH-resembling phenotype comprising fibrosis, tubular dilation and thickening of tubular basement membranes. Additional knockout of YAP rescued this kidney phenotype (Xu et al., 2020), further indicating that targeting of the Hippo pathway could be an interesting approach for NPH.

##### Wnt inhibition

Another key developmental pathway is the Wingless-Int-1 (Wnt) pathway, although the role of cilia in canonical, β-catenin-dependent Wnt signaling remains controversial (Wheway et al., 2018). Proteins from the InvsC disrupted in infantile NPH including INVS/NPHP2 and NPHP3 have been proposed to be required for the switch from canonical Wnt signaling, involved in cell proliferation, to non-canonical Wnt signaling which maintains planar cell polarity, although the precise role in cystogenesis is debated (Simons et al., 2005; Bergmann et al., 2008; Sugiyama et al., 2011; Wang et al., 2018). Planar cell polarity is essential for oriented cell division which controls tubule elongation and is impaired in most murine renal ciliopathy models (Fischer and Pontoglio, 2009). Interestingly, RPGRIP1L and NPHP4 were also involved in Wnt signaling through their functional interaction with INVS (Burckle et al., 2011; Mahuzier et al., 2012).

Increased canonical Wnt signaling has been suggested to induce cystogenesis in PKD and NPH (Malik et al., 2020). Knockdown of *DCDC2/NPHP19* has been shown to activate the canonical Wnt pathway and cause pronephric cyst formation in zebrafish, a phenotype which could be rescued by a β-catenin inhibitor (Schueler et al., 2015).

#### Targeting Cilia-Independent Signaling Pathways and Cell Cycle Regulation

##### CDKs

Specific ciliary proteins are involved in DDR signaling and consequent cell cycle regulation. Although drugs that interfere directly with DDR signaling have not been studied in this context, the dysregulated cell cycle has been investigated as a target for the treatment of cysts and fibrosis in renal ciliopathies (Ibraghimov-Beskrovnaya, 2007; Choi et al., 2013).

Administration of the broad CDK inhibitor roscovitine (which targets CDK1, CDK2, CDK5 and CDK7, arresting the cell cycle) effectively arrested kidney volume expansion, cyst progression and renal insufficiency in *jck/Nek8/Nphp9* and *cpk* (cystin) mouse models of infantile NPH and PKD, respectively, with long-lasting effects. Analysis of the targets of roscovitine revealed induction of a G1/S cell-cycle block, transcriptional regulation and inhibition of apoptosis as mechanisms of action (Bukanov et al., 2006). In addition to the mouse models, roscovitine treatment also showed partial rescue of pronephric cysts and improved renal filtration capacity in zebrafish morphants of ciliopathy-associated genes (Tobin and Beales, 2008). Another study in *jck/Nek8/Nphp9* mice demonstrated that roscovitine ameliorated the elongated cilia phenotype and restored tubular epithelial differentiation, while conditional inactivation of CDK5 reduced cilia length, total kidney volume and cyst formation (Husson et al., 2016). In contrast, *Cdk2*-deficient *jck/Nek8/Nphp9* mice showed no improvement of renal cyst growth, inflammation and fibrosis, likely due to compensatory upregulation of *Cdk1*. In addition, treatment with the mTOR inhibitor rapamycin reduced Cdk1 and Cdk2 activity and attenuated the cystic phenotype, suggesting that the effect of mTOR inhibition is partly mediated by CDK inhibition (Zhang et al., 2020). Finally, roscovitine was demonstrated to attenuate renal cyst progression in a kidney-specific *Cep164*-knockout mouse model characterized by rapidly progressive cystic kidney enlargement (Airik et al., 2019). In summary, studies performed mostly in models of infantile NPH have demonstrated that targeting cell cycle dysregulation and proliferation using CDK inhibitors is an efficient approach to limit cyst growth. Of note, part of the effects of roscovitine, and also of its derivative CR8, could however be linked to its potent inhibition of Casein Kinase 1 family members which expression pattern is perturbed in cystic mouse model including infantile NPH models (*pcy/Nphp3*, *Jck/Nek8/Nphp9)* (Billot et al., 2018). Interestingly, this study also indicates that the expression of the cell cycle regulator p21cip1/WAF1, which inhibits CDK2, is decreased in all cystic models while it is increased in the kidneys of *Nphp4* mutant mice, again suggesting differential pathophysiological mechanisms between cystic infantile and juvenile forms of NPH.

Interestingly, targeting of CDK1/2 was also shown to be effective in a *Cep290/Nphp6* mouse model characterized by slowly progressive kidney disease. Treatment of kidney cells from *Cep290-*deficient mice with CDK1/2 inhibitor rescued DNA-damage signaling, supernumerary centrioles and ciliation defects (Slaats et al., 2015). Furthermore, knockdown of CDK5 in URECs from a patient with Joubert syndrome with compound heterozygous mutations in *CEP290* mitigated the elongated cilia phenotype observed in these cells. In addition, treatment of the cells with the Hh agonist purmorphamine reduced expression of CDK5, suggesting a convergence of the Hh and CDK signaling pathways (Srivastava et al., 2017).

##### Calmodulin and Ca^2+^/calmodulin-dependent protein kinase II

Ca^2+^/calmodulin-dependent protein kinase II (CaMKII) is an important mediator of the endoplasmic reticulum stress response, oxidative stress and the mitochondrial apoptotic pathway, which were shown to be upregulated in kidneys from *jck/Nek8/Nphp9* mice. Pharmacological inhibition of CaMKII restored these pathways and significantly reduced kidney volume and cystogenesis (Bracken et al., 2016). CaMKII additionally has a role in cell cycle progression (Skelding et al., 2011). CaMKII inhibition could therefore also reduce cystogenesis through a mechanism similar to CDK inhibitors. Interestingly, calmodulin is known to bind to IQCB1/NPHP5 (Otto et al., 2005) and was recently shown to negatively regulate the amount of NPHP5 present at the transition zone (Kim et al., 2018). Eupatilin, a compound able to rescue ciliogenesis in *CEP290* invalidated cells (see below), directly modulates the calmodulin/NPHP5 interaction therefore increasing the amount of NPHP5 at the transition zone in the absence of CEP90 (Kim et al., 2018). Interestingly, Eupatilin was recently shown to improve cilia-related phenotypes in the context of *RPGRIP1L/NPHP8* suggesting that it might present broader potential in transition zone-related NPH (Wiegering et al., 2021).

##### ERK and p38-MAPK inhibition

The ERK pathway (downstream of receptor tyrosine kinases and GPCR signaling), is involved in regulation of cell cycle and proliferation and was shown to be activated as a consequence of increased cAMP in ADPKD (Yamaguchi et al., 2003). Inhibition of ERK activation slowed down disease progression in *pcy/Nphp3* mice (Omori et al., 2006). It also resulted in decreased fibrosis and cystogenesis in an *Invs/Nphp2* mouse model (Okumura et al., 2009). The same group later showed that a p38 mitogen-activated protein kinase (MAPK) inhibitor reduced renal fibrosis but not cystogenesis independently of ERK activation, however, this did not improve kidney function and survival rate of *Invs* mice (Sugiyama et al., 2012).

#### Inflammation and Senescence

In addition to ciliary signaling pathways and cystogenesis, specific interventions directly target development of tubulointerstitial fibrosis, a hallmark histopathological feature of juvenile and adult NPH. The role of inflammation in fibrosis has been discussed in section “Inflammation and Senescence.” Several groups have targeted the immune response in NPH. For example, rodent models of infantile NPH (Han:SPRD-*Cy/Anks6/Nphp16* rats and *pcy/nphp3* mice) treated with the anti-inflammatory drug methylprednisolone showed reduced fibrosis and preserved kidney function compared to untreated animals (Gattone et al., 1995). Treatment of Han:SPRD-*Cy/Anks6/Nphp16* rats with the anti-inflammatory drug resveratrol attenuated cyst formation, elevated serum creatinine and macrophage infiltration partly through inhibition of mTOR signaling and the NF-kB pathway (Wu et al., 2016). Furthermore, a study using LPK*/Nek8/Nphp9* mutant rats found that treatment with the immunomodulatory drug dimethyl fumarate, which activates the Nrf2 pathway and inhibits NF-kB signaling, reduced macrophage infiltration but did not ameliorate cyst progression or renal insufficiency (Oey et al., 2018).

Replicative senescence in tubular cells, which leads to secretion of pro-inflammatory molecules, has been shown to cause extensive kidney damage in *Glis2/Nphp7* knockout mice (Jin et al., 2020) (see section “Pathophysiology of Nephronophthisis”). The senolytic (anti-aging) drug forkhead box protein O4 D-retro inverso (FOXO4-DRI), which induces apoptosis of senescent cells through inhibition of FOXO4/P53 interaction (Baar et al., 2017), effectively eliminated senescent cells in mouse models for acute kidney disease and reduced inflammation and tubulointerstitial fibrosis while preserving renal function in *Glis2*-knockout mice (Jin et al., 2019, 2020). The role of senescence and the efficacy of senolytic drugs in other types of NPH is unknown and should be investigated further.

#### Glycosphingolipids and Cholesterol

Several studies demonstrated that glycosphingolipid metabolism is perturbed in PKD (Deshmukh et al., 1994; Chatterjee et al., 1996). Interestingly, glycosphingolipids are components of rafts, membrane microdomains involved in the assembly of signaling platforms at the plasma membrane that are also present at the ciliary membrane. Dysregulated production of those lipids may therefore contribute to perturbed signaling and therefore to cystogenesis (Natoli et al., 2020). Interestingly, glucosylceramides, which are glycosphingolipid precursors, were found to be highly increased in the kidneys of cystic mouse models including in *pcy/Nphp3* and *Jck/Nek8/Nphp9* mice and pharmacological or genetic inhibition of glucosylceramide synthase activity efficiently reduced renal cysts in *Jck/Nek8/Nphp9* mice (Natoli et al., 2010, 2012).

Besides glycosphingolipids, cholesterol is also involved in many cellular functions including in signaling through Ras and plasma membrane lipid rafts (Ahmadi et al., 2020). Cholesterol synthesis can be modulated by statins, a family of molecules which have been widely used to treat hypercholesterolemia. In addition to its classical well-established functions, cholesterol is enriched in the ciliary membrane and was revealed to play an important role in ciliogenesis (Maerz et al., 2019) as well as in Hh signaling as a direct modulator of SMO activity (Kong et al., 2019). Interestingly, and in agreement with those observations, mutations in genes encoding actors of the cholesterol biosynthesis pathway were associated with skeletal disorders (Suzuki et al., 2020) resembling those associated with mutations in IFT-A subunit encoding genes (see introduction). Statins were shown to improve CKD in various animal models (Ecder, 2016) and were also shown to improve renal function in the *Cy/Anks6/Nphp16* rat model, likely through an effect on the renin-angiotensin system (Gile et al., 1995; Zafar et al., 2007). Several studies obtained conflicting results on the beneficial effects of statins in PKD patients (Fassett et al., 2010; Cadnapaphornchai et al., 2014; Brosnahan et al., 2017; Müller and Schermer, 2019). It is currently not known if cholesterol dysregulation is associated with NPH or if modulating cholesterol synthesis could be an interesting approach in the context of NPH other than in infantile NPH rodent models.

#### Microtubule Dynamics

Cytoplasmic microtubule dynamics directly influences ciliogenesis (Sharma et al., 2011) and their reorganization tightly controls polarity establishment in epithelia (Toya and Takeichi, 2016). Interestingly, ciliopathy conditions were associated with increased stability of cytoplasmic microtubules (Berbari et al., 2013), a phenotype which was more deeply investigated in the context of NPH and *TRAF3IP1/IFT54* mutant conditions (Berbari et al., 2011; Bizet et al., 2015). Impaired IFT54 function led to overexpression of the microtubule associated protein MAP4 and lowering MAP4 expression rescued polarity defects *in vitro* and partially rescued ciliopathy phenotypes in zebrafish morphants (Bizet et al., 2015). It would be interesting to investigate if stabilization of the microtubules is a phenotype shared with other NPHP defects. These results indicate that modulators of cytoplasmic microtubule dynamics could be a potential therapeutic approach for NPH.

Interestingly, expression of HDAC6 was found to be increased in various kidney conditions including fibrotic, ADPKD and CKD models, and HDAC6 inhibitors showed a positive effect on disease evolution (Ke et al., 2018). HDAC6 is a histone deacetylase which is key regulator of the cytoskeleton through deacetylation of α-tubulin and cortactin. HADC6 was also found to be involved in cilia disassembly through destabilization of axonemal microtubules (Pugacheva et al., 2007; Loktev et al., 2008). Functions of HDAC6 are wide and complex, however, and its overexpression likely yields deacetylation which is expected to result in destabilization of microtubules (contrary to increased stability of microtubules observed in *TRAF3IP1/IFT54* mutant conditions). This suggests differential microtubule involvement between NPH and other renal disorders.

### Drug Screens for Nephronophthisis

Pharmacological screens for ciliopathies comprise small molecule compound screens and high-throughput testing of chemical libraries such as United States Food and Drug Administration (FDA)-approved drugs with the aim of drug repurposing. The latter is an attractive option because it is cheaper, faster, and has a higher success rate than development of novel drugs (Forsythe et al., 2018). Thus far, compound screens in cystic renal disease have mostly been performed for ADPKD, the most common ciliopathy affecting the kidney (Booij et al., 2017, 2019). High-throughput drug screens for NPH-related ciliopathies are scarce in the literature and findings have not been replicated (Kim and Kim, 2019).

One of the few published compound screens was conducted by Kim et al. (2018), who screened a library of 2,789 synthetic and natural compounds in *CEP290*-invalidated human RPE1 cells and identified 22 compounds that restored ciliogenesis, of which the flavonoid eupatilin showed the strongest effect. Eupatilin was demonstrated to restore IQCB1/NPHP5 levels at the ciliary transition zone in the absence of CEP290 (see above). Eupatilin treatment did not significantly rescue ciliogenesis defects associated with the knockdown of other NPH-associated genes, including *NPHP4*, *CEP164/NPHP15* and *CEP83/NPHP18*, suggesting a *CEP290*-specific effect. Treatment of rd16 mice harboring a *Cep290* in-frame deletion with eupatilin improved cone photoreceptor function, showing its efficacy *in vivo* (Kim et al., 2018). However, because this *Cep290* mouse model of Leber congenital amaurosis (LCA) does not present a kidney phenotype (Chang et al., 2006), the effect of eupatilin on NPH was not investigated.

Two compound screens were performed in zebrafish models. A library of 115 compounds targeting cilia specific pathways was screened in *pkd2-* and *ift172*-morphant embryos which present pronephric cysts (Cao et al., 2009). A histone deacetylase (HDAC) inhibitor was identified that suppressed cyst formation in *pkd2*- but not in *ift172*-morphants, making this finding less relevant for NPH. Cox2-inhibitors and a CaMKII inhibitor were identified to affect extrarenal ciliopathy phenotypes in both models (Cao et al., 2009). In addition, an automated imaging pipeline was recently developed to efficiently profile a large compound library in *ift172*-morphant zebrafish embryos, however, identified pronephric cyst-modifying compounds were not reported (Pandey et al., 2019).

Another strategy that has been employed is an untargeted small interfering RNA (siRNA) screen to identify genes that modulate ciliogenesis and can be targeted pharmacologically. For example, an siRNA screen in RPE1 cells targeting 7,784 therapeutically relevant genes identified 36 positive modulators of ciliogenesis including proteins involved in the regulation of actin cytoskeleton dynamics. Pharmacological inhibition of actin polymerization using cytochalasin D rescued ciliogenesis in *Ift88*-mutant mouse embryonic fibroblasts (Kim et al., 2010). However, cytochalasin D is likely not specific enough for treatment of ciliopathies. In addition, a recent genome-wide siRNA screen identified 10 genes that rescued aberrant Wnt signaling in *BBS4*-depleted cells (Tsai et al., 2019). One of the identified genes encoded USP35, a negative regulator of the ubiquitin proteasome system. Suppression of USP35 improved the kidney phenotype in *bbs4*-morphant zebrafish embryos, suggesting that impaired clearance of signaling molecules could play a role in NPH pathophysiology. Next, a small molecule inhibitor of another deubiquitinase (USP14; IU1) was tested in zebrafish and did not have a significant effect on the kidney phenotype, consistent with the results from the screen (Tsai et al., 2019). High-throughput phenotype-based screening methods using zebrafish and cell models have been outlined for renal ciliopathies (Gehrig et al., 2018; Pandey et al., 2019; Zhang P. et al., 2019).

## Gene-Based Therapy

A promising gene-based strategy makes use of antisense-oligonucleotides (ASO) to modulate splicing. This approach was first employed to correct a recurrent intronic *CEP290/NPHP6* mutation present in 10–15% of LCA cases, which introduces a cryptic splice site leading to inclusion of a pseudo exon with a premature stop codon (Collin et al., 2012). ASO targeting of this mutation successfully restored mRNA splicing, CEP290 expression and cilia in patient cells and patient iPSC-derived retinal organoids (Gerard et al., 2012; Parfitt et al., 2016) and led to vision improvement in LCA patients (Cideciyan et al., 2019). ASO was also used to induce skipping of nonsense mutation-bearing exons as well as treat non-retinal manifestations. For example, systemic ASO treatment of a *Cep290* mouse model of Joubert syndrome resulted in reduced kidney cyst burden compared to untreated mice (Ramsbottom et al., 2018). While *CEP290* is unique in that it contains numerous in-frame exons that can be targeted by ASO, basal exon skipping has also been shown to modify the phenotype in *CC2D2A*-related Joubert syndrome and could be induced in other ciliopathy-associated genes (Drivas et al., 2015; Ramsbottom et al., 2018). Although ASO cannot be applied in case of a homozygous *NPHP1* deletion which is the most common cause of NPH, it can be used to target downstream dysregulated genes as was demonstrated for *Agt* encoding angiotensinogen in the treatment of *Pkd1* mice (Saigusa et al., 2016; Fitzgibbon et al., 2018). An advantage of ASO-based treatment for NPH is that circulating ASO are cleared by and accumulate in the kidney where they are taken up especially but not exclusively by tubular cells, facilitating delivery to target tissue (Oberbauer et al., 1996; Lakhia et al., 2019). Research into ligand-based targeted ASO delivery is ongoing (Kim and Kim, 2019).

Additionally, CRISPR/Cas9 has shown promising results for the treatment of NPH in *in vitro* systems. In contrast to classical gene therapy in which a wild-type copy of the gene is integrated into the host genome, CRISPR/Cas9 is a precise genome-editing technique that allows use of a repair template. This correction approach is widely used to obtain isogenic control iPSCs and it was shown to efficiently restore the cilia phenotype in iPSC-derived kidney organoids from a patient with *IFT140*-related Mainzer-Saldino syndrome. In addition, spheroids from epithelial cells sorted from uncorrected organoids were less polarized than spheroids from gene-corrected cells (Forbes et al., 2018). However, implementation in patients with renal ciliopathies is met by technical challenges (for example off-target effects, delivery to the kidney and evading an immune response) and ethical obstacles. In addition, the extreme genetic heterogeneity in NPH-related ciliopathies, i.e., the absence of mutation hot spots except for the homozygous *NPHP1* deletion, and possible role of modifier variants, limit the practical and financial feasibility of gene-targeted therapies (Kim and Kim, 2019).

## Developments and Future Directions

Nephronophthisis is an important hereditary cause of ESRD in children and young adults for which there is currently no targeted therapy. Pharmacological interventions aimed at restoring aberrant signal transduction (for example V2R antagonists) or dysregulated cell cycle (for example CDK inhibitors) as well as the use of immunomodulatory drugs (for example senolytic drugs) have improved cystic phenotypes in zebrafish and rodent models of NPH (Gattone et al., 2003; Bukanov et al., 2006; Jin et al., 2020). In addition, ASO therapy partly restored gene expression and reversed cyst burden in a *Cep290* mouse model of Joubert syndrome (Ramsbottom et al., 2018). ASO therapy has been approved by the FDA for clinical use in several human diseases including spinal muscular atrophy and Duchenne muscular dystrophy, suggesting that application in NPH is feasible (Fay et al., 2020). However, the *Cep290* mouse model, like most rodent and zebrafish models reviewed here, does not exhibit the massive tubulointerstitial fibrosis observed in NPH patients, which severely limits conclusions about efficacy of potential therapy for juvenile NPH (Hynes et al., 2014; Ramsbottom et al., 2018).

Truly representative models for juvenile NPH are not available yet except for *Glis2/Nphp7* knockout mice, and *GLIS2* mutations are an atypical and rare cause of NPH (Lu et al., 2016). In fact, most mouse models used in research on NPH therapy correspond genetically and phenotypically mainly to infantile NPH, which represents a minor fraction of NPH patients, is histologically different from the more prevalent juvenile NPH and shows a more rapid disease course (see introduction).

Alternative non-orthologous animal models that mimic juvenile/adult NPH could also be explored. For example, mice with kidney-specific knockout of *Lkb1* showed a urine concentration defect, diminished kidney size, cysts at the corticomedullary junction, tubular basement membrane thickening, inflammation and fibrosis (Han et al., 2016; Viau et al., 2018). Interestingly, LKB1 (STK11) was shown to be part of a ciliary module comprising NPHP1, NEK7, ANKS3 and polycystin-1 that regulates cilia-controlled secretion of CCL2, a chemokine that promotes macrophage recruitment and consequent cyst growth and interstitial inflammation (Viau et al., 2018). In addition, mice with kidney-specific knockout of *Prkar1a*, a regulatory subunit of PKA, presented with normal-sized kidneys with small cysts and interstitial fibrosis (Ye et al., 2017), suggesting that cAMP signaling defects not only play a role in cystogenesis but also in the development of juvenile NPH-specific phenotypes. Kidney-specific knockout of *Aatf* (DNA damage response target Apoptosis Antagonizing Transcription Factor), encoding a regulator of p53 during DDR, also leads to phenotypes similar to juvenile NPH including tubular atrophy, interstitial fibrosis and cysts at the corticomedullary junction (Jain et al., 2019). Lastly, *Fan1* knockout mice treated with cisplatin showed a histological phenotype that included tubular dilations, tubular basement membrane thickening and interstitial fibrosis, resembling juvenile NPH except for the presence of karyomegalic nuclei (Zhou et al., 2012; Airik et al., 2016). These models might point to key signaling pathways and might prove more useful for testing fibrosis-modulating drugs for juvenile NPH than genetically identical models.

During the past 5 years, an increasing number of pharmacological studies in NPH have focused on fibrosis-associated pathways and fibrosis-modulating agents. Drugs that interfere with cell cycle progression such as CDK inhibitors typically affect ubiquitous and essential cellular processes, which limits their application in NPH treatment. Alternatively, the pro-inflammatory response downstream of cell cycle arrest can be targeted to prevent tubular damage and fibrosis (Jin et al., 2020). Specifically, the role of cellular senescence and efficacy of senolytic drugs remains to be investigated in prevalent NPH subtypes. Of note, the CDK inhibitor roscovitine was shown to promote apoptosis of neutrophils and enhance resolution of inflammation, indicating crosstalk between both mechanisms (Rossi et al., 2006). Additional progress can be gained from repurposing of medication approved for other fibrotic kidney diseases, for example COX inhibitors which have shown potential in the prevention of diabetic nephropathy in addition to ameliorating NPH in rodent models (Goru, 2018).

Despite *in vitro* and *in vivo* evidence for drugs that ameliorate (part of) the NPH phenotype, there is a striking lack of clinical trials for NPH. To exclude possible publication bias, ClinicalTrials.gov was queried for registered ongoing and future clinical trials in ciliopathies. This yielded trials for ADPKD, ARPKD, primary ciliary dyskinesia, Von Hippel Lindau disease and various extrarenal manifestations of ciliopathies, including obesity in Bardet-Biedl and Alström syndromes (clinicaltrials.gov – access date 2021 April 12). There are several possible reasons for the absence of clinical trials in NPH, including challenges related to the genetic and phenotypic heterogeneity in NPH, for example small sample sizes and variability within cohorts, the relatively short therapeutic window compared to ADPKD, challenges of clinical trials in a pediatric population and financial aspects of orphan drug development.

In light of this, research groups should capitalize on available data in renal ciliopathies and investigate associations between medication for (extra)renal symptoms and fluctuation in renal phenotype, for example statins which can be prescribed to treat hypercholesterolemia in patients with Bardet-Biedl or Alström syndrome have shown promising but inconsistent results in ADPKD (Fassett et al., 2010; Cadnapaphornchai et al., 2014; Brosnahan et al., 2017; Müller and Schermer, 2019). In addition, data should be collected to gain insight into ciliopathy-specific symptomatic treatment. For example, in a patient with *NPHP3*-related infantile NPH isosorbide dinitrate was used to treat hypertension by restoring nitric oxide generation (Strong et al., 2018). Nitric oxide is produced by vascular endothelial cells in response to ciliary sensation of shear stress and causes compensatory vasodilatation, a process that is impaired in ciliopathies. Targeting this cilia-specific cause of hypertension was more effective in this patient than classic anti-hypertensive medication (Strong et al., 2018).

Another consequence of the low prevalence and phenotypic heterogeneity in NPH is the need for personalized therapy. Developments in *in vitro* modeling, for example the use of URECs or the generation of kidney organoids from iPSCs, enable testing of medication directly in NPH patient-derived cells that harbor the causal mutation and the genetic background of the patient including modifier gene variants. This should facilitate translation of findings back to therapy in individual patients. Despite current limitations, improvement of NPH-kidney organoids can aid drug development in NPH, as has been successfully illustrated in a kidney organoid model of cystinosis (Hollywood et al., 2020).

Patient-derived organoids can be combined with CRISPR/Cas9 technology to correct the NPH phenotype for various applications (Steichen et al., 2020). To overcome technical and ethical hurdles associated with *in vivo* therapeutic application of CRISPR/Cas9, genome editing can be applied *ex vivo*, for example to populate bioartificial kidneys with corrected patient-derived renal epithelial cells as a future alternative for donor kidneys if safety issues of cell-based therapies have been sufficiently resolved (McKee and Wingert, 2016; Mihajlovic et al., 2019a, b). Bioengineering strategies in kidney regeneration have been reviewed by Peired et al. (2020).

Besides *ex vivo* applications, developments in targeted delivery methods bring gene therapy in NPH patients one step closer (Liu et al., 2019). Whereas adeno-associated virus (AAV)-vector delivery of wildtype gene and CRISPR/Cas9 to retinal cells via subretinal injection has been demonstrated in *Bbs4* and *Cep290* mouse models, respectively, delivery of viral vector to the kidney via systemic injection is limited because AAV particles do not pass the glomerular filtration barrier (Simons et al., 2011; Ruan et al., 2017; Rubin et al., 2019). Targeted delivery and nephron segment-specific gene expression has been achieved via retrograde ureteral infusion of AAV9 vector in combination with segment-specific promoters in mice (Asico et al., 2018). This system of targeted delivery could be used for gene-based therapy in NPH. In addition, systemic administration of (artificial) extracellular vesicles or nanoparticles has been proposed for targeted delivery of siRNA or drugs to kidney cells (Barile and Vassalli, 2017; Oroojalian et al., 2020).

## Conclusion

Many potential therapies for NPH have been explored in model systems, from targeted treatments aimed at restoring ciliary signaling, CDK inhibition, immune system suppression and ASO therapy to untargeted drug screens. Translation of results is limited by the lack of animal models that recapitulate the juvenile NPH phenotype and in the case of infantile NPH, for which models exist, by the short therapeutic window. Employment of non-orthologous animal models and developments in organoid technology can potentially fill this gap and provide new opportunities for personalized treatments. Ultimately, safety and efficacy of potential NPH therapies will have to be tested in clinical trials. Given obstacles of genetic and phenotypic heterogeneity in renal ciliopathies and clinical trials in children, repurposing of medication used in other cystic or chronic kidney diseases could be the most viable approach. In addition to identification of effective drugs, successful treatment or prevention of NPH depends on the discovery of biomarkers that identify NPH patients in an early disease stage.

## Author Contributions

MS and AB conceived the idea and wrote the manuscript. AB and SS provided critical feedback. All authors contributed to the article and approved the submitted version.

## Conflict of Interest

The authors declare that the research was conducted in the absence of any commercial or financial relationships that could be construed as a potential conflict of interest.
